# Fly Transmission of *Campylobacter*

**DOI:** 10.3201/eid1103.040460

**Published:** 2005-03

**Authors:** Gordon L. Nichols

**Affiliations:** * Health Protection Agency, London, United Kingdom

**Keywords:** Campylobacter, Climate, diarrhea, disease outbreaks, Epidemiologic Methods, Diptera, Seasons, Houseflies, Disease Transmission

An annual increase in *Campylobacter* infection in England and Wales begins in May and reaches a maximum in early June. This increase occurs in all age groups and is seen in all geographic areas. Examination of risk factors that might explain this seasonal increase identifies flies as a potential source of infection. The observed pattern of infection is hypothesized to reflect an annual epidemic caused by direct or indirect contamination of people by small quantities of infected material carried by flies that have been in contact with feces. The local pattern of human illness appears random, while having a defined geographic and temporal distribution that is a function of the growth kinetics of one or more fly species. The hypothesis provides an explanation for the seasonal distribution of *Campylobacter* infections seen around the world.

*Campylobacter* spp. are the most common bacterial causes of diarrhea in England and Wales (1). The epidemiologic features of *Campylobacter* infection have proved difficult to discover, and extensive strain typing has failed to clarify the main transmission routes. Testable hypotheses must be established to explain available evidence, particularly the reason for the observed seasonality. Relatively few outbreaks of *Campylobacter* gastroenteritis occur (2), and most cases are sporadic. In case-control and case-case studies of sporadic *Campylobacter* infections, most cases remain unexplained by recognized risk factors (3,4).

The annual increase in *Campylobacter* infections in England and Wales begins at approximately day 130 (May 9) and reaches a maximum at approximately day 160 (June 8) (Figure 1). Although this seasonal rise is seen in all ages, it is more marked in children (5). Cases in towns and cities across England and Wales show broadly similar seasonal changes in distribution (Figure 2). The relative geographic uniformity of the increase seen in May of most years has the temporal appearance of an annual national epidemic. Because person-to-person infection within the community is uncommon, it is likely that the epidemic is caused by a single main driver for human *Campylobacter* infection. The possible seasonal drivers were examined, and only vector transmission by flies appears to provide a convincing explanation for the observed seasonal trends (Table).

**Figure 1:**
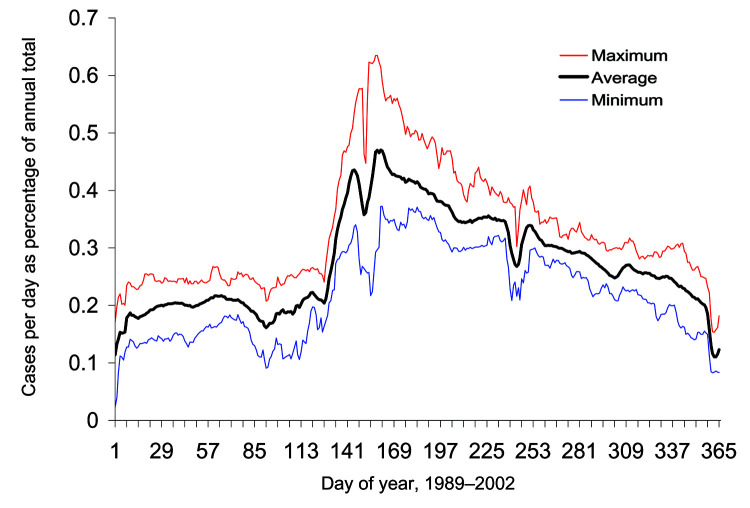
Distribution of Campylobacter cases per day. When averaged for 1989 to 2002, the epidemic begins at approximately day 130, peaks at approximately day 160, and gradually declines through the rest of the year.

**Figure 2:**
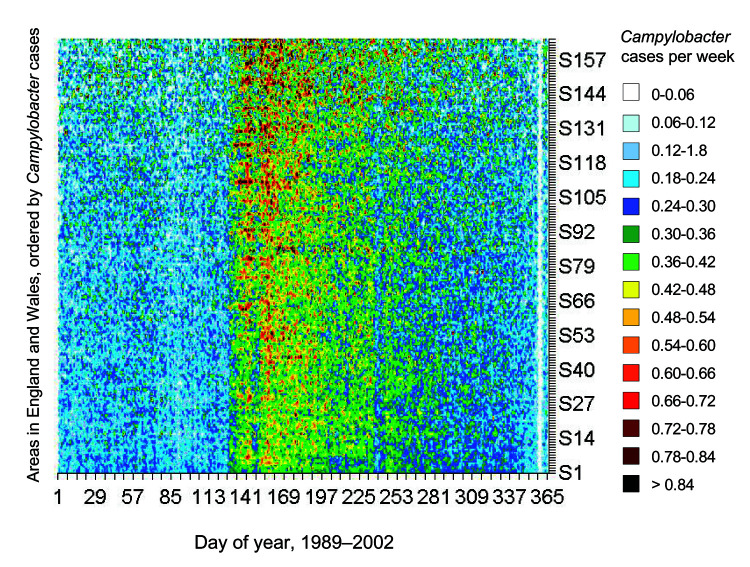
Cases of Campylobacter infection in England and Wales based on the patient specimen date. Figure shows broadly similar changes in patterns of infection across the country as measured by laboratory reporting per town or city (cases as a percentage of the annual total) by day of year. Laboratories were ordered by the total number of cases reported over the 14-year period (Appendix).

The seasonal increase in *Campylobacter* infections in May and June in England and Wales is hypothesized to reflect an annual epidemic caused by direct or indirect exposure of humans to contaminated material carried by several fly species that have been in contact with human, bird, or animal feces or contaminated raw foods. Flies have been shown to carry *Campylobacter* and can infect both humans and animals (6–8). Intervention studies have demonstrated diarrheal disease reduction linked to control of flies (9–11), and deaths from diarrheal diseases have been linked to measurements of fly abundance (12). The local pattern of human *Campylobacter* infection appears random, while having a defined geographic and temporal distribution. This distribution is predicted to be linked to the growth kinetics of 1 or more fly species and their access to environmental sources of *Campylobacter* in feces or food. The seasonal increase in fly populations results from rainy weather and an increase in temperature that causes the development from egg to fly to occur in days rather than months. Individual flies can lay hundreds of eggs, which can result in a large increase in fly numbers in a short period. Fly numbers fluctuate through the summer and decline in October, but the decline is less dramatic and defined than the spring increase.

Disease transmission is hypothesized to occur through small quantities of contaminated material carried on the feet, proboscis, legs, and body hairs or from material regurgitated or defecated by flies. The variety, numbers, virulence and viability of organisms in the contaminated material will differ, and some contamination will include *Campylobacter* while others will not. Contamination will be distributed over a variety of food types. Contamination of food by flies could occur at any stage of the food supply chain, but *Campylobacter* counts within the contaminated material on foods will decrease over time; consequently, most infection will result from contamination close to consumption (e.g., in the domestic or catering environment). Because whether a fly has visited contaminated feces is unknown and how a person becomes infected is uncertain, epidemiologic investigation is difficult.

A number of synanthropic fly species could be involved, including houseflies (e.g., *Musca* spp., *Fannia* spp.), blowflies (e.g., *Calliphora* spp., *Lucilia* spp.), and other dung-related flies (e.g., *Sarcophaga* spp., *Drosophila* spp.) (13). These flies have individual behavioral patterns, ecology, physiology, and temporal and geographic distributions that will influence the likelihood of their being in kitchens, on human or animal feces, and on food. Although *Musca domestica* is the species most likely to be involved because it is commonly found in houses and food-processing establishments, larger flies (e.g., *Calliphora* spp.) may be able to transmit larger numbers of *Campylobacter*.

Flies contaminated through fecal contact will carry heterogeneous mixtures of organisms, including any pathogens that are present within the feces, and may be able to cause a variety of human infections, including infection by different *Campylobacter* species and types. This fact partially explains the lack of a clear epidemiologic picture arising from *Campylobacter* typing work. Gastrointestinal disease caused by flies is more likely to involve pathogens with a low infectious dose (e.g., *Shigella*, *Campylobacter*, *Cryptosporidium*, *Giardia*, *Cyclospora*, *Escherichia coli* O157), and some of these could have a seasonal component related to flies. Where high fly populations and poor hygiene conditions prevail, as in disasters or famines, or where pathogens can grow within fly-contaminated food, the potential exists for transmitting pathogens with a high infectious dose (e.g., *Vibrio cholerae*, *Salmonella* spp.). The access that flies have to human and animal feces will influence the degree to which they are contaminated with different enteric pathogens.

Contamination of a range of foods by flies will result in a pattern of infection that will not be amenable to identifying specific vehicles through standard case-control, case-case, or cohort studies, unless specific objective or subjective assessments of fly numbers can be obtained. Fly monitoring will need to be undertaken. An alternative approach could use estimates of fly population numbers based on climatic conditions to compare with data on human *Campylobacter* infections. This approach has the advantage of being able to use historical climatic and disease surveillance data. The broad relationship between *Campylobacter* cases and ambient temperature has not been explained in terms of disease causation. The time taken for the larvae of *M. domestica* to develop (13) was applied to temperature data for England and Wales and has been used to show a strong relationship between *Campylobacter* cases per week and *M. domestica* larval development time for 1989 to 1999 (Figure 3). Periods when *Campylobacter* cases exceed a 7-day average of 170 cases per day occurred when *M. domestica* larval development time was <3 weeks.

**Figure 3:**
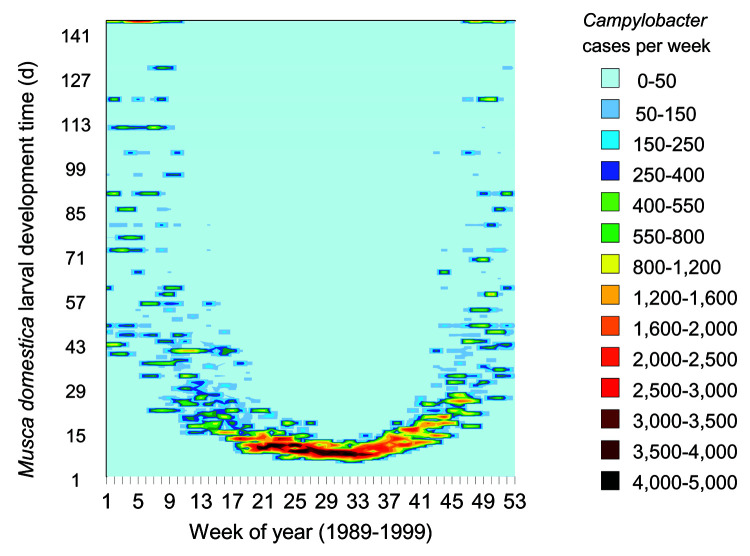
Campylobacter cases by week and Musca domestica larval growth times. Campylobacter cases per day are plotted against the minimum M. domestica growth times for the 14 days before the date for weeks from January 1989 to December 1999. The time taken for M. domestica larvae to develop was based on understood growth temperatures (145 days divided by the number of degrees above 12°C, up to an optimum of 36°C) (8). The temperatures were based on a maximum temperature in 47 temperature sampling sites across England and Wales in the 2 weeks before (Appendix).

The hypothesis predicts that the *Campylobacter* infection rates will be higher in persons living close to animal production and lower in urban settings because fly numbers will be lower. Some evidence from the United Kingdom (1,14) and Norway (15) supports this hypothesis. Seasonal changes in *Campylobacter* incidence that are seen around the world may result from changes in fly populations and flies' access to human and animal feces. Much emphasis on foodborne disease reduction has rightly been on kitchen hygiene, since the low infectious dose of *Campylobacter* makes cross-transmission from raw meats to ready-to-eat foods a substantial risk in domestic and catering environments. Fly transmission may be the most important source of infection in kitchen transmission routes, and establishments that sell ready-to-eat foods may be sources of *Campylobacter*, if effective fly control is not in operation. Flies may also be important in transmitting *Campylobacter* in poultry flocks (16) and between other agricultural animals.

While flies are regarded as important mechanical vectors of diarrheal disease in developing countries, control has largely concentrated on improving drinking water and sewage disposal. In the industrialized world, flies are thought to play a minor role in the transmission of human diarrheal diseases. Immediately intervening in the transmission of *Campylobacter* gastroenteritis should be possible through increased public awareness and more effective fly control.

## Appendix

### Supplementary Information

### Temperature Data

**Figure A1:**
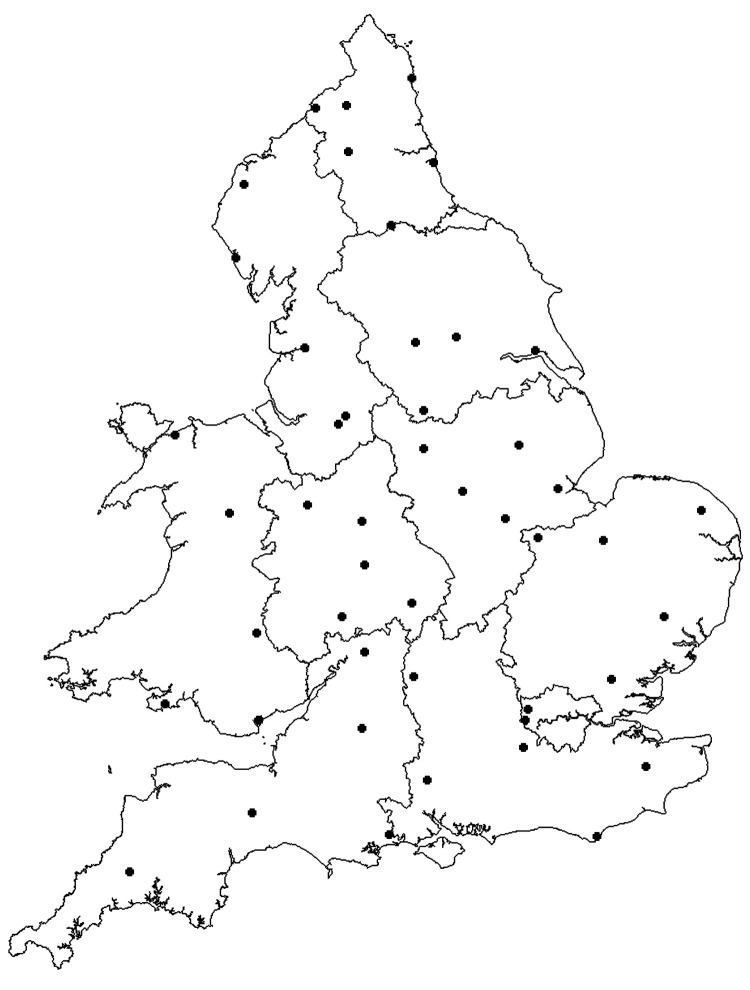
Temperature station locations.

Temperature data were acquired from the British Atmospheric Data Centre (BADC), the Natural Environment Research Council's (NERC) Designated Data Centre for the Atmospheric Sciences based at the Rutherford Appleton Laboratory in Oxfordshire, part of the Central Laboratory of the Research Councils. Data are available on-line through a World Wide Web interface (http://badc.nerc.ac.uk) by prearranged agreement. Data were collated for the period 1989–1999, with 5 locations selected for each region to provide overall coverage of the region (except London, which had only 2 centers with data available for the given time period). Location of temperature stations is shown in the Figure A1.

There were a total of 47 sites. Some of the data series were missing data points. The maximum, minimum, and average temperatures were determined for all days between January 1, 1989, and December 31, 1999. Maximum temperatures across all sites were used to calculate the presumptive minimum *Musca domestica* larval development times.

### Methods

The data represent patients who had fecal specimens examined by a microbiology laboratory in England and Wales between 1989 and 2003 where *Campylobacter* was isolated from the sample. Data were acquired through well-described surveillance processes, and analysis was conducted in Microsoft Access and Excel (Microsoft Corp., Redmond, WA, USA). Daily cases were based on the patient specimen date, and a 7-day rolling mean was used to eliminate the weekly cycles that reflect reduced patient sampling on weekends.

Hypothesis generation was performed through a systematic review of known and suggested causes of *Campylobacter* infection, particularly reflecting on changes in these risks over the period of May and June and assessing their credibility as biological drivers for the observed seasonality.

### Cities and Towns Included in Figure A1

S1, London; S2, Birmingham; S3, Bristol; S4, Nottingham; S5, Sheffield; S6, Manchester; S7, Leeds; S8, Leicester; S9, Reading; S10, Plymouth; S11, Portsmouth; S12, Colchester; S13, Bradford; S14, Southampton; S15, Poole; S16, Preston; S17, Cardiff; S18, Chelmsford; S19, Norwich; S20, Ipswich; S21, Truro; S22, Oxford; S23, Shrewsbury; S24, Dudley; S25, Taunton; S26, Newport; S27, Cambridge; S28, Newcastle; S29, Chester; S30, Gloucester; S31, Swindon; S32, Chertsey; S33, Coventry; S34, Welwyn; S35, Frimley Park; S36, High Wycombe; S37, Slough; S38, Exeter; S39, Swansea; S40, Luton; S41, Torquay; S42, Derby; S43, York; S44, Worcester; S45, Northampton; S46, Bishops Stortford; S47, Hull; S48, Basildon; S49, Stoke-on-Trent; S50, Worthing; S51, Stafford; S52, Harrogate; S53, Hereford; S54, Halifax; S55, Sunderland; S56, Chesterfield and N Derbyshire; S57, Lincoln; S58, Ashford Kent; S59, Stockport; S60, Blackpool; S61, Maidstone; S62, Liverpool; S63, Bangor; S64, Llandough; S65, Lancaster; S66, Sutton Coldfield; S67, Aylesbury; S68, Grimsby; S69, Doncaster; S70, Peterborough; S71, Brighton; S72, Gateshead; S73, Kettering; S74, Southend; S75, Rhyl; S76, Cheltenham; S77, Epsom; S78, Chichester; S79, Carlisle; S80, Milton Keynes; S81, Dorchester; S82, Durham; S83, Bury; S84, Great Yarmouth; S85, Bury St Edmunds; S86, Warwick; S87, Salisbury; S88, Wolverhampton; S89, Scarborough; S90, Pontefract; S91, Bath; S92, Winchester; S93, Bishop Auckland; S94, Watford; S95, Bolton; S96, Eastbourne; S97, Oldham; S98, North Shields; S99, Burnley; S100, Ashford Middlesex; S101, Kings Lynn; S102, Warrington; S103, Wakefield; S104, Keighley; S105, Crawley; S106, Barnstaple; S107, Abergavenney; S108, Boston; S109, Nuneaton; S110, Northallerton; S111, Wrexham; S112, Macclesfield; S113, Darlington; S114, Bedford; S115, Basingstoke; S116, Weston Supermare; S117, Middlesborough; S118, Dewsbury; S119, Sutton-in-Ashfield; S120, Rochdale; S121, Guildford; S122, Worksop; S123, Wigan; S124, Stevenage; S125, Bridgend; S126, Rotherham; S127, West Bromwich; S128, Solihull; S129, Burton-upon-Trent; S130, Haverford West; S131, Carmarthen; S132, Hemel Hempstead; S133, Stockton-on-Tees; S134, Huddersfield; S135, South Shields; S136, Barnsley; S137, Whitehaven; S138, Chatham; S139, Blackburn; S140, Redditch; S141, St Leonards-on-Sea; S142, Grantham and Kesteven; S143, Ormskirk; S144, Scunthorpe; S145, Canterbury; S146, Kidderminster; S147, Dartford; S148, Aberystwyth; S149, Hexham; S150, Barrow-in Furness; S151, Redhill; S152, Margate; S153, Walsall; S154, Ashington; S155, Salford; S156, Merthyr Tydfil; S157, Stourbridge; S158, Haywards Heath; S159, Banbury; S160, Hartlepool; S161, Prescot; S162, Otley; S163, Southport; S164, Yeovil; S165, Llanelli. The number of reported *Campylobacter* cases per city and town were based on reports from all laboratories serving the area and are ordered from highest (S1) to lowest (S165) case numbers. Results from towns reporting smaller numbers of cases were excluded from the analysis.

### Factors Linked to *Campylobacter* Infection

The Table A1 provides evidence for seasonal associations between factors linked to human *Campylobacter* infections or outbreaks.

## Supplementary Material

Table A1 Table A1 in PDF format.

## Appendix

**Table:** Risk factors that might affect *Campylobacter* seasonality*

Risk factor	Outbreaks	Evidence of seasonality	Credibility as the main seasonal driver
Barbecuing	Yes	Medium	Low
Birds	Yes	Strong	Low
Bottled water	No	None	Low
Chicken	Yes	Medium	Medium
Cross-contamination	Yes	None	None
Domestic catering	No	None	None
Farm visit	Yes	None	None
Farm animals	Yes	Weak	Low
Flies	No	Strong	High
Food handlers	Yes	None	None
Food packaging	No	None	None
Immunologic response	No	Weak	None
Mains supply drinking water	Yes	None	None
Nosocomial	Yes	None	None
Pets	No	Weak	Low
Pools, lakes, streams	No	None	None
Private drinking water supplies	Yes	Weak	None
Protozoa	No	None	Low
Salads and fruit	Yes	Weak	Low
Stir-fried food	Yes	None	None
The countryside	No	Weak	Medium
Transmission in families	Yes	None	None
Travel abroad	No	None	None
Unpasteurized milk	Yes	Weak	None
Weather/climate	No	Medium	Medium

*Evidence base provided in Appendix.

**Table A1:** Evidence for seasonal associations between factors linked to human *Campylobacter* infections or outbreaks. Download PDF(71Kb, 6 pages).

Risk factor	Outbreaks	Evidence for factor causing seasonal increase	Evidence against factor causing seasonal increase
Chicken/turkey	(17–23)	Chicken is the food most commonly contaminated with *Campylobacter*. A substantial portion of infection probably derives from this source (17–22,24–26). Some evidence shows that *Campylobacter* contamination of chickens is seasonal.	Chicken is not the vehicle for most sporadic *Campylobacter* infections (24,27,28). Little evidence exists that the seasonal differences in *Campylobacter* in chickens are sufficient to drive the seasonality of human disease (29-34).
Salads and fruit	(35-37)	Untreated leaf salads and soft fruits might be potential sources of human campylobacteriosis (25, 35-37) because these raw products are eaten without any heat treatment.	In most of the outbreaks involving salad items, cross-contamination from contaminated raw foods was thought to be involved. While seasonal import of fresh fruit or vegetables from different countries might represent a potential source of infection it would be surprising if this manifested itself as an annual nationwide outbreak across the whole of England and Wales while remaining refractory to epidemiologic investigation. Fly transmission from animal feces may be important.
Cross-contamination from raw meats to ready-to-eat foods	(25)	Cross-contamination from raw meats to ready to eat foods within kitchens and retail premises probably contributes significantly to *Campylobacter* infection.	Why cross-contamination should be strongly influenced by the season is unclear, unless levels of raw meat contamination change with the seasons.
Unpasteurized or inadequately pasteurized milk	(22, 38-49)	Unpasteurized or badly pasteurized milk can be a source of *Campylobacter* infection (22,39, 42, 45, 49-52). Milk could cause the seasonality if the numbers of *Campylobacter* in raw milk changed with the season and other critical control points in milk production (pasteurization) are not tightly maintained. Infections related to consumption of unpasteurized milk appear to be seasonal, with a peak in May, which suggests seasonal changes in the *Campylobacter* contamination of unpasteurized milk.	No evidence shows that the seasonality of human disease is largely due to unpasteurized milk because this product is not commonly consumed. No evidence shows that pasteurization varies substantially by season.
Birds	(53,54)	*Campylobacter* is common in birds. Migratory birds result in large seasonal changes in the inputs to the environment from bird feces and could contribute to human *Campylobacter* exposure (55). Migratory birds could be a seasonally changing driver to human disease (56). The main likely exposure route if this were the case would be direct contact with contaminated bird feces in the garden, contamination of field-grown fruit and vegetables and contamination of source waters for drinking. Bird-pecked milk is a recognized route by which *Campylobacter* infection can be acquired (53,54). The contamination is thought to result from birds feeding consecutively on cow feces and milk in bottles. The infections related to bird-pecked milk appear to be seasonal in distribution with a marked increase in May (57).	Bird-pecked milk is unlikely to be the cause of the worldwide seasonal distribution of *Campylobacter* infections. Fly transmission from bird feces, particularly farmed poultry, may be important. Evidence from extensive monitoring of ready-to-eat foods sampled at retail businesses suggests little evidence of *Campylobacter* contamination (Little, pers. comm.).
Barbecue	(17)	Barbecue use might be a contributing factor to the total *Campylobacter* infection because standards of food safety associated with barbecue use are likely to be poorer (17,58, 59). Case-control studies have found associations between barbecue use and sporadic *Campylobacter* infection (60,61).	Barbecue use on its own is unlikely a big enough, or seasonal enough, driver of disease to account for seasonal changes in incidence.
Food packaging		The packaging around chickens is commonly contaminated with *Campylobacter*, which may represent a source of some infections through cross-contamination.	Strong seasonal changes in the extent of this contamination would have to exist for this factor to affect the disease epidemiology, and no evidence for these changes exists.
Food handlers/hygiene	(62-66)	Infected food handlers might represent a source of infection in catering premises.	Infections in food handlers probably are seasonal, reflecting the seasonality of *Campylobacter* in general, but they are probably not the driver for the overall seasonality.
Food, stir-fried	(17)	Stir-fried food may be contaminated through inadequately cooking raw ingredients or cross-contamination.	A seasonal change in the contamination of raw ingredients would need to exist to explain the epidemiology.
Flies		Flies provide a biological explanation for the spring increase in *Campylobacter* cases through the increase in fly numbers. *Campylobacter* has been isolated from flies, and the low infectious dose required to cause human disease would make this route credible. Historical records link “summer diarrhea” to flies.	Little hard evidence exists for this transmission route.
Mains drinking water	(44, 67-76)		With mains water supplies, the relatively even distribution of seasonal changes in the distribution of *Campylobacter* cases suggests that any contamination of public supplies must be systemic (a generic problem with all supplies) or a much bigger regional difference in the incidence would be seen. Potential seasonal differences in water quality that could explain why treatment might not prevent sporadic *Campylobacter* infection through mains water (e.g., viable noncultivable *Campylobacter* in chlorine-resistant protozoa) are not supported by evidence. The rarity of outbreaks associated with public water supplies suggests that drinking water is not a substantial source of *Campylobacter* infection.
Private drinking water supplies/untreated surface water, rain water, or well water	(22, 75,77-86)	Waterborne infection associated with private water supplies can result in outbreaks of infection because many people drink the contaminated water (87). *Campylobacter* is the most common organism causing these outbreaks. A seasonal change in water quality could occur.	Seasonal changes in water contamination should trigger outbreaks rather than a national increase in sporadic disease. The comparative rarity of outbreaks associated with private supplies suggests that this source does not substantially contribute to the total illness that is seen to change dramatically with the season. Given the influence of surface water on the microbiologic quality of private water supplies, we expect that the seasonal occurrence of *Campylobacter* might be more influenced by rainfall than time of year, which does not appear to happen.
Bottled water		In a case-case study of *Campylobacter*, people with *C. coli* infection were more likely to have drunk bottled water than were those with *C. jejuni* infection (88). Natural mineral water is not disinfected and could be a widely dispersed product that experiences seasonal changes in contamination.	Sources of water that are used to produce natural mineral water and other bottled waters are relatively well protected. These groundwaters are unlikely to be contaminated with *Campylobacter*. If bottled water consumption is a risk factor, it should come up as such in analytic epidemiologic studies of *Campylobacter* infection. It is unclear why the seasonal pattern of infection should be so constant both geographically and annually if bottled water contamination is such a substantial contributor to human disease.
Pools, lakes, and streams		Potential exists for illness after swallowing contaminated recreational water (89-92). Water sports in natural waters can be a source of exposure. If the contamination of water with *Campylobacter* is seasonal, then any seasonality in this group could be linked to either changes in water quality or behavior.	Illness associated with recreational water activity has not been established, and this is unlikely to be the source of the spring increase in campylobacteriosis. Little evidence shows that the change in recreational water activity in the spring is enough to explain the seasonal change in *Campylobacter* cases.
Within-family transmission	(93)	Person-to-person transmission can occur.	No obvious reason explains why within-household transmission of *Campylobacter* should be seasonal, given that personal hygiene practices are not likely to change substantially over a matter of weeks.
Domestic catering		Domestic food preparation may contribute to human *Campylobacter* disease.	Fly transmission within kitchens may contribute to transmission, and this would likely be seasonal. Little else within the kitchen environment, other than the contamination of raw food ingredients, is likely to vary seasonally.
Nursery/childcare/school	(94,95)	As *Campylobacter* is common in children, transmission may occur within the childcare setting.	No evidence shows that infections in childcare are common or that they vary through the year.
Nosocomial transmission	(96)		Nosocomial transmission cannot account for the national seasonal increase in cases.
Pets		Pets, particularly kittens and puppies, have been postulated as a source of *Campylobacter*. Canine births, as recorded in Kennel Club and Guide Dogs for the Blind Association records, show a strong seasonal distribution, and this factor has been proposed as a driver for human disease (97).	Little evidence shows that the seasonal change in *Campylobacter* is directly related to pets, although fly transmission from animal feces may be important.
Farm animals	(98)	*Campylobacter* strains isolated from cattle have been linked to strains from human infections (99,100). Cattle and sheep represent a reservoir of *Campylobacter* (101,102), and milkborne outbreaks (2^3^, ^39^,^42^,^45^,^49^-^55^) suggest that other routes may occur. Fecal shedding by sheep may be more frequent around lambing (103). Seasonal differences in *Campylobacter* infections have also been demonstrated in rhesus monkeys, other agricultural animals, and birds (31, 32, 104-107).	Any seasonality of *Campylobacter* infection or colonization in animals could cause seasonality in humans, but this seasonality is most likely to result from the contamination of food. Fly transmission from animal feces may be important.
Farm visits	(108)	Visits to farms can expose children to common zoonotic enteric pathogens, including *Campylobacter*.	Any seasonality of farm visits is unlikely to contribute to the seasonal distribution of all cases.
The countryside		Direct environmental exposure could occur through walking in the country.	This activity may be seasonal but is unlikely to contribute to the strong seasonal distribution of cases.
Travel		*Campylobacter* has been linked to overseas travel (109-111), including military service (112,113), and probably represents a significant percentage of all cases of travelers' diarrhea (114-117). In some countries, >50% of *Campylobacter* cases may be linked to foreign travel (118)	The seasonality of *Campylobacter* does not follow the seasonality of travel abroad.
Weather/climate		In some developing countries a higher incidence was seen in the rainy season (119, 120), which suggests flies might be contributory. Although *Campylobacter* is more common during the summer months and has been linked to temperature (121), no direct relationship was seen between temperature and cases of human disease. The different seasonal distribution in different countries appears to be partly temperature-related	Little evidence shows that *Campylobacter* is associated with rainfall. There was no association between thermophilic *Campylobacter* in lambs at slaughter and rainfall (105). The main seasonal driver for *Campylobacter* infection is not likely to be rainfall itself, since the increase appears to occur annually, irrespective of when most rain falls.
Immunologic response		The immunologic response to *Campylobacter* exposure could change throughout the year. This hypothesis has been studied in male rhesus monkeys (104). A marked seasonality was seen ,with the frequency of TH1-type cytokine synthesis in the summer being markedly greater than in the winter, whereas TH2-type cytokine expression did not vary between the seasons.	Current evidence suggests that seasonal changes in immunologic response to *Campylobacter* infection are unlikely to account for the major seasonal changes in *Campylobacter* incidence.
