# Supplementary material for: Fly Transmission of Campylobacter
Source: Emerg Infect Dis. 2005 Mar;11(3):361–4. doi: 10.3201/eid1103.040460 (PMC3298251; doi:10.3201/eid1103.040460)
Supplement: Table A1 — in PDF format. [file 04-0460-TA1.pdf]

Nichols GL. Fly transmission of *Campylobacter*. Emerg Infect Dis [serial on the Internet]. 2005 Mar [*date cited*]. Available from http://www.cdc.gov/ncidod/EID/vol11no03/04-0460.htm

Appendix Table. Evidence for seasonal associations between factors linked to human *Campylobacter* infections or outbreaks

**Risk factor**	**Outbreaks**	**Evidence for factor causing seasonal increase**	**Evidence against factor causing seasonal increase**
Chicken/turkey	(1–7)	Chicken is the food most commonly contaminated with *Campylobacter.* A substantial portion of infection probably derives from this source (1–6,8–10). Some evidence shows that *Campylobacter* contamination of chickens is seasonal.	Chicken is not the vehicle for most sporadic *Campylobacter* infections (8,11,12). Little evidence exists that the seasonal differences in *Campylobacter* in chickens are sufficient to drive the seasonality of human disease (13–18).
Salads and fruit	(19–21)	Untreated leaf salads and soft fruits might be potential sources of human campylobacteriosis (9,19–21) because these raw products are eaten without any heat treatment.	In most of the outbreaks involving salad items, cross-contamination from contaminated raw foods was thought to be involved. While seasonal import of fresh fruit or vegetables from different countries might represent a potential source of infection it would be surprising if this manifested itself as an annual nationwide outbreak across the whole of England and Wales while remaining refractory to epidemiologic investigation. Fly transmission from animal feces may be important.
Cross-contamination from raw meats to ready-to-eat foods	(9)	Cross-contamination from raw meats to ready to eat foods within kitchens and retail premises probably contributes significantly to *Campylobacter* infection.	Why cross-contamination should be strongly influenced by the season is unclear, unless levels of raw meat contamination change with the seasons.
Unpasteurized or inadequately pasteurized milk	(6,22–33)	Unpasteurized or badly pasteurized milk can be a source of *Campylobacter* infection (6,23,26,29,33–36). Milk could cause the seasonality if the numbers of *Campylobacter* in raw milk changed with the season and other critical control points in milk production (pasteurization) are not tightly maintained. Infections related to consumption of unpasteurized milk appear to be seasonal, with a peak in May, which suggests seasonal changes in the *Campylobacter* contamination of unpasteurized milk.	No evidence shows that the seasonality of human disease is largely due to unpasteurized milk because this product is not commonly consumed. No evidence shows that pasteurization varies substantially by season.
Birds	(37,38)	*Campylobacter* is common in birds. Migratory birds result in large seasonal changes in the inputs to the environment from bird feces and could contribute to human *Campylobacter* exposure (39). Migratory birds could be a seasonally changing driver to human disease (40). The main likely exposure route if this were the case would be direct contact with contaminated bird feces in the garden, contamination of field-grown fruit and vegetables and contamination of source waters for drinking. Bird-pecked milk is a recognized route by which *Campylobacter* infection can be acquired (37,38). The contamination is thought to result from birds feeding consecutively on cow feces and milk in bottles. The infections related to bird-pecked milk appear to be seasonal in distribution with a marked increase in May (41).	Bird-pecked milk is unlikely to be the cause of the worldwide seasonal distribution of *Campylobacter* infections. Fly transmission from bird feces, particularly farmed poultry, may be important. Evidence from extensive monitoring of ready-to-eat foods sampled at retail businesses suggests little evidence of *Campylobacter* contamination (Little, pers. comm.).
Barbecue	(1)	Barbecue use might be a contributing factor to the total *Campylobacter* infection because standards of food safety associated with barbecue use are likely to be poorer (1,42,43). Case-control studies have found associations between barbecue use and sporadic *Campylobacter* infection (44,45).	Barbecue use on its own is unlikely a big enough, or seasonal enough, driver of disease to account for seasonal changes in incidence.

Risk factor	Outbreaks	Evidence for factor causing seasonal increase	Evidence against factor causing seasonal increase
Food packaging		The packaging around chickens is commonly contaminated with *Campylobacter*, which may represent a source of some infections through cross-contamination.	Strong seasonal changes in the extent of this contamination would have to exist for this factor to affect the disease epidemiology, and no evidence for these changes exists.
Food handlers/hygiene	(46–50)	Infected food handlers might represent a source of infection in catering premises.	Infections in food handlers probably are seasonal, reflecting the seasonality of *Campylobacter* in general, but they are probably not the driver for the overall seasonality.
Food, stir-fried	(2)	Stir-fried food may be contaminated through inadequately cooking raw ingredients or cross-contamination.	A seasonal change in the contamination of raw ingredients would need to exist to explain the epidemiology.
Flies		Flies provide a biological explanation for the spring increase in *Campylobacter* cases through the increase in fly numbers. *Campylobacter* has been isolated from flies, and the low infectious dose required to cause human disease would make this route credible. Historical records link “summer diarrhea” to flies.	Little hard evidence exists for this transmission route.
Mains drinking water	(28,51–60)		With mains water supplies, the relatively even distribution of seasonal changes in the distribution of *Campylobacter* cases suggests that any contamination of public supplies must be systemic (a generic problem with all supplies) or a much bigger regional difference in the incidence would be seen. Potential seasonal differences in water quality that could explain why treatment might not prevent sporadic *Campylobacter* infection through mains water (e.g., viable noncultivable *Campylobacter* in chlorine-resistant protozoa) are not supported by evidence. The rarity of outbreaks associated with public water supplies suggests that drinking water is not a substantial source of *Campylobacter* infection.
Private drinking water supplies/untreated surface water, rain water, or well water	(6,59;61–70)	Waterborne infection associated with private water supplies can result in outbreaks of infection because many people drink the contaminated water (71). *Campylobacter* is the most common organism causing these outbreaks. A seasonal change in water quality could occur.	Seasonal changes in water contamination should trigger outbreaks rather than a national increase in sporadic disease. The comparative rarity of outbreaks associated with private supplies suggests that this source does not substantially contribute to the total illness that is seen to change dramatically with the season. Given the influence of surface water on the microbiologic quality of private water supplies, we expect that the seasonal occurrence of *Campylobacter* might be more influenced by rainfall than time of year, which does not appear to happen.
Bottled water		In a case-case study of *Campylobacter*, people with *C. coli* infection were more likely to have drunk bottled water than were those with *C. jejuni* infection (72). Natural mineral water is not disinfected and could be a widely dispersed product that experiences seasonal changes in contamination.	Sources of water that are used to produce natural mineral water and other bottled waters are relatively well protected. These groundwaters are unlikely to be contaminated with *Campylobacter*. If bottled water consumption is a risk factor, it should come up as such in analytic epidemiologic studies of *Campylobacter* infection. It is unclear why the seasonal pattern of infection should be so constant both geographically and annually if bottled water contamination is such a substantial contributor to human disease.
Pools, lakes, and streams		Potential exists for illness after swallowing contaminated recreational water (73–76). Water sports in natural waters can be a source of exposure. If the contamination of water with *Campylobacter* is seasonal, then any seasonality in this group could be linked to either changes in water quality or behavior.	Illness associated with recreational water activity has not been established, and this is unlikely to be the source of the spring increase in campylobacteriosis. Little evidence shows that the change in recreational water activity in the spring is enough to explain the seasonal change in *Campylobacter* cases.

Risk factor	Outbreaks	Evidence for factor causing seasonal increase	Evidence against factor causing seasonal increase
Within-family transmission	(77)	Person-to-person transmission can occur.	No obvious reason explains why within-household transmission of *Campylobacter* should be seasonal, given that personal hygiene practices are not likely to change substantially over a matter of weeks.
Domestic catering		Domestic food preparation may contribute to human *Campylobacter* disease.	Fly transmission within kitchens may contribute to transmission, and this would likely be seasonal. Little else within the kitchen environment, other than the contamination of raw food ingredients, is likely to vary seasonally.
Nursery/childcare/school	(78,79)	As *Campylobacter* is common in children, transmission may occur within the childcare setting.	No evidence shows that infections in childcare are common or that they vary through the year.
Nosocomial transmission	(80)		Nosocomial transmission cannot account for the national seasonal increase in cases.
Pets		Pets, particularly kittens and puppies, have been postulated as a source of *Campylobacter*. Canine births, as recorded in Kennel Club and Guide Dogs for the Blind Association records, show a strong seasonal distribution, and this factor has been proposed as a driver for human disease (81).	Little evidence shows that the seasonal change in *Campylobacter* is directly related to pets, although fly transmission from animal feces may be important.
Farm animals	(82)	*Campylobacter* strains isolated from cattle have been linked to strains from human infections (83,84). Cattle and sheep represent a reservoir of *Campylobacter* (85,86), and milkborne outbreaks (6,23,26,29,33–36) suggest that other routes may occur. Fecal shedding by sheep may be more frequent around lambing (87). Seasonal differences in *Campylobacter* infections have also been demonstrated in rhesus monkeys, other agricultural animals, and birds (15,16,88–91).	Any seasonality of *Campylobacter* infection or colonization in animals could cause seasonality in humans, but this seasonality is most likely to result from the contamination of food. Fly transmission from animal feces may be important.
Farm visits	(92)	Visits to farms can expose children to common zoonotic enteric pathogens, including *Campylobacter*.	Any seasonality of farm visits is unlikely to contribute to the seasonal distribution of all cases.
The countryside		Direct environmental exposure could occur through walking in the country.	This activity may be seasonal but is unlikely to contribute to the strong seasonal distribution of cases.
Travel		*Campylobacter* has been linked to overseas travel (93–95), including military service (96,97), and probably represents a significant percentage of all cases of travelers' diarrhea (98–101). In some countries, >50% of *Campylobacter* cases may be linked to foreign travel (102)	The seasonality of *Campylobacter* does not follow the seasonality of travel abroad.
Weather/climate		In some developing countries a higher incidence was seen in the rainy season (103,104), which suggests flies might be contributory. Although *Campylobacter* is more common during the summer months and has been linked to temperature (105), no direct relationship was seen between temperature and cases of human disease. The different seasonal distribution in different countries appears to be partly temperature-related	Little evidence shows that *Campylobacter* is associated with rainfall. There was no association between thermophilic *Campylobacter* in lambs at slaughter and rainfall (89). The main seasonal driver for *Campylobacter* infection is not likely to be rainfall itself, since the increase appears to occur annually, irrespective of when most rain falls.
Immunologic response		The immunologic response to *Campylobacter* exposure could change throughout the year. This hypothesis has been studied in male rhesus monkeys (88). A marked seasonality was seen ,with the frequency of TH1-type cytokine synthesis in the summer being markedly greater	Current evidence suggests that seasonal changes in immunologic response to *Campylobacter* infection are unlikely to account for the major seasonal changes in *Campylobacter* incidence.

Risk factor	Outbreaks	Evidence for factor causing seasonal increase	Evidence against factor causing seasonal increase
		than in the winter, whereas TH2-type cytokine expression did not vary between the seasons.	

Appendix References

1. Allerberger F, Al Jazrawi N, Kreidl P, Dierich MP, Feierl G, Hein I, et al. Barbecued chicken causing a multi-state outbreak of *Campylobacter jejuni* enteritis. Infection. 2003;31:19–23.
2. Evans MR, Lane W, Frost JA, Nylen G. A campylobacter outbreak associated with stir-fried food. Epidemiol Infect. 1998;121:275–9.
3. Kessel AS, Gillespie IA, O'Brien SJ, Adak GK, Humphrey TJ, Ward LR. General outbreaks of infectious intestinal disease linked with poultry, England and Wales, 1992–1999. Commun Dis Public Health. 2001;4:171–7.
4. Murphy O, Gray J, Gordon S, Bint AJ. An outbreak of campylobacter food poisoning in a health care setting. J Hosp Infect. 1995;30:225–8.
5. Pearson AD, Greenwood MH, Donaldson J, Healing TD, Jones DM, Shahamat M, et al. Continuous source outbreak of campylobacteriosis traced to chicken. J Food Prot. 2000;63:309–14.
6. Pebody RG, Ryan MJ, Wall PG. Outbreaks of campylobacter infection: rare events for a common pathogen. Commun Dis Rep CDR Rev. 1997;7:R33–7.
7. Shandera WX, Tormey MP, Blaser MJ. An outbreak of bacteremic *Campylobacter jejuni* infection. Mt Sinai J Med. 1992;59:53–6.
8. Rodrigues LC, Cowden JM, Wheeler JG, Sethi D, Wall PG, Cumberland P, et al. The study of infectious intestinal disease in England: risk factors for cases of infectious intestinal disease with *Campylobacter jejuni* infection. Epidemiol Infect. 2001;127:185–93.
9. Centers for Disease Control and Prevention. Outbreak of *Campylobacter* enteritis associated with cross-contamination of food—Oklahoma, 1996. MMWR Morb Mortal Wkly Rep. 1998;47:129–31.
10. Layton MC, Calliste SG, Gomez TM, Patton C, Brooks S. A mixed foodborne outbreak with *Salmonella heidelberg* and *Campylobacter jejuni* in a nursing home. Infect Control Hosp Epidemiol. 1997;18:115–21.
11. Neal KR, Slack RC. Diabetes mellitus, anti-secretory drugs and other risk factors for campylobacter gastro-enteritis in adults: a case-control study. Epidemiol Infect. 1997;119:307–11.
12. Adak GK, Cowden JM, Nicholas S, Evans HS. The Public Health Laboratory Service national case-control study of primary indigenous sporadic cases of campylobacter infection. Epidemiol Infect. 1995;115:15–22.
13. Wilson IG. Salmonella and campylobacter contamination of raw retail chickens from different producers: a six-year survey. Epidemiol Infect. 2002;129:635–45.
14. Hanninen ML, Perko-Makela P, Pitkala A, Rautelin H. A three-year study of *Campylobacter jejuni* genotypes in humans with domestically acquired infections and in chicken samples from the Helsinki area. J Clin Microbiol. 2000;38:1998–2000.
15. Hudson JA, Nicol C, Wright J, Whyte R, Hasell SK. Seasonal variation of *Campylobacter* types from human cases, veterinary cases, raw chicken, milk and water. J Appl Microbiol. 1999;87:115–24.
16. Wallace JS, Stanley KN, Currie JE, Diggle PJ, Jones K. Seasonality of thermophilic *Campylobacter* populations in chickens. J Appl Microbiol. 1997;82:219–24.
17. Humphrey TJ, Henley A, Lanning DG. The colonization of broiler chickens with *Campylobacter jejuni*: some epidemiological investigations. Epidemiol Infect. 1993;110:601–7.
18. Kapperud G, Skjerve E, Vik L, Hauge K, Lysaker A, Aalmen I, et al. Epidemiological investigation of risk factors for campylobacter colonization in Norwegian broiler flocks. Epidemiol Infect. 1993;111:245–55.
19. Kirk M, Waddell R, Dalton C, Creaser A, Rose N. A prolonged outbreak of *Campylobacter* infection at a training facility. Commun Dis Intell. 1997;21:57–61.
20. Roels TH, Wickus B, Bostrom HH, Kazmierczak JJ, Nicholson MA, Kurzynski TA, et al. A foodborne outbreak of *Campylobacter jejuni* (O:33) infection associated with tuna salad: a rare strain in an unusual vehicle. Epidemiol Infect. 1998;121:281–7.
21. Ronveaux O, Quoilin S, Van Loock F, Lheureux P, Struelens M, Butzler JP. A *Campylobacter coli* foodborne outbreak in Belgium. Acta Clin Belg. 2000;55:307–11.
22. Centers for Disease Control and Prevention. Outbreak of *Campylobacter jejuni* infections associated with drinking unpasteurized milk procured through a cow-leasing program—Wisconsin, 2001. MMWR Morb Mortal Wkly Rep. 2002;51:548–9.
23. Evans MR, Roberts RJ, Ribeiro CD, Gardner D, Kembrey D. A milk-borne campylobacter outbreak following an educational farm visit. Epidemiol Infect. 1996;117:457–62.
24. Fahey T, Morgan D, Gunneburg C, Adak GK, Majid F, Kaczmarski E. An outbreak of *Campylobacter jejuni* enteritis associated with failed milk pasteurisation. J Infect. 1995;31:137–43.
25. Jones PH, Willis AT, Robinson DA, Skirrow MB, Josephs DS. Campylobacter enteritis associated with the consumption of free school milk. J Hyg (Lond). 1981;87:155–62.
26. Kalman M, Szollosi E, Czermann B, Zimanyi M, Szekeres S, Kalman M. Milkborne campylobacter infection in Hungary. J Food Prot. 2000;63:1426–9.
27. Korlath JA, Osterholm MT, Judy LA, Forfang JC, Robinson RA. A point-source outbreak of campylobacteriosis associated with consumption of raw milk. J Infect Dis. 1985;152:592–6.
28. Lind L, Sjogren E, Melby K, Kaijser B. DNA fingerprinting and serotyping of *Campylobacter jejuni* isolates from epidemic outbreaks. J Clin Microbiol. 1996;34:892–6.
29. Morgan D, Gunneberg C, Gunnell D, Healing TD, Lamerton S, Soltanpoor N, et al. An outbreak of *Campylobacter* infection associated with the consumption of unpasteurised milk at a large festival in England. Eur J Epidemiol. 1994;10:581–5.
30. Porter IA, Reid TM. A milk-borne outbreak of *Campylobacter* infection. J Hyg (Lond). 1980;84:415–9.
31. Robinson DA, Edgar WJ, Gibson GL, Matchett AA, Robertson L. Campylobacter enteritis associated with consumption of unpasteurised milk. Br Med J. 1979;1:1171–3.
32. Robinson DA, Jones DM. Milk-borne campylobacter infection. Br Med J (Clin Res Ed). 1981;282:1374–6.
33. Wood RC, MacDonald KL, Osterholm MT. Campylobacter enteritis outbreaks associated with drinking raw milk during youth activities. A 10-year review of outbreaks in the United States. JAMA. 1992;268:3228–30.
34. Lehner A, Schneck C, Feierl G, Pless P, Deutz A, Brandl E, et al. Epidemiologic application of pulsed-field gel electrophoresis to an outbreak of *Campylobacter jejuni* in an Austrian youth centre. Epidemiol Infect. 2000;125:13–6.

35. Djuretic T, Wall PG, Nichols G. General outbreaks of infectious intestinal disease associated with milk and dairy products in England and Wales: 1992 to 1996. Commun Dis Rep CDR Rev. 1997;7:R41–5.
36. Public Health Laboratory Service Study Group. Cryptosporidiosis in England and Wales: prevalence and clinical and epidemiological features. BMJ. 1990;300:774–7.
37. Riordan T, Humphrey TJ, Fowles A. A point source outbreak of campylobacter infection related to bird-pecked milk. Epidemiol Infect. 1993;110:261–5.
38. Stuart J, Sufi F, McNulty C, Park P. Outbreak of campylobacter enteritis in a residential school associated with bird-pecked bottle tops. Commun Dis Rep CDR Rev. 1997;7:R38–40.
39. Waldenstrom J, Broman T, Carlsson I, Hasselquist D, Achterberg RP, Wagenaar JA, et al. Prevalence of *Campylobacter jejuni*, *Campylobacter lari*, and *Campylobacter coli* in different ecological guilds and taxa of migrating birds. Appl Environ Microbiol. 2002;68:5911–7.
40. Broman T, Palmgren H, Bergstrom S, Sellin M, Waldenstrom J, Danielsson-Tham ML, et al. *Campylobacter jejuni* in black-headed gulls (*Larus ridibundus*): prevalence, genotypes, and influence on *C. jejuni* epidemiology. J Clin Microbiol. 2002;40:4594–602.
41. Sopwith W, Ashton M, Frost JA, Tocque K, O'Brien S, Regan M, et al. Enhanced surveillance of campylobacter infection in the north west of England 1997–1999. J Infect. 2003;46:35–45.
42. Butzler JP, Oosterom J. Campylobacter: pathogenicity and significance in foods. Int J Food Microbiol. 1991;12:1–8.
43. Kapperud G. Campylobacter infection. Epidemiology, risk factors and preventive measures. Tidsskr Nor Laegeforen. 1994;114:795–9.
44. Ikram R, Chambers S, Mitchell P, Brieseman MA, Ikam OH. A case control study to determine risk factors for campylobacter infection in Christchurch in the summer of 1992–3. N Z Med J. 1994;107:430–2.
45. Kapperud G, Skjerve E, Bean NH, Ostroff SM, Lassen J. Risk factors for sporadic *Campylobacter* infections: results of a case-control study in southeastern Norway. J Clin Microbiol. 1992;30:3117–21.
46. Fitzgerald C, Helsel LO, Nicholson MA, Olsen SJ, Swerdlow DL, Flahart R, et al. Evaluation of methods for subtyping *Campylobacter jejuni* during an outbreak involving a food handler. J Clin Microbiol. 2001;39:2386–90.
47. Olsen SJ, Hansen GR, Bartlett L, Fitzgerald C, Sonder A, Manjrekar R, et al. An outbreak of *Campylobacter jejuni* infections associated with food handler contamination: the use of pulsed-field gel electrophoresis. J Infect Dis. 2001;183:164–7.
48. Gent RN, Telford DR, Syed Q. An outbreak of campylobacter food poisoning at a university campus. Commun Dis Public Health. 1999;2:39–42.
49. Wight JP, Rhodes P, Chapman PA, Lee SM, Finner P. Outbreaks of food poisoning in adults due to *Escherichia coli* O111 and campylobacter associated with coach trips to northern France. Epidemiol Infect. 1997;119:9–14.
50. Winquist AG, Roome A, Mshar R, Fiorentino T, Mshar P, Hadler J. Outbreak of campylobacteriosis at a senior center. J Am Geriatr Soc. 2001;49:304–7.
51. Alary M, Nadeau D. An outbreak of *Campylobacter* enteritis associated with a community water supply. Can J Public Health. 1990;81:268–71.
52. Engberg J, Gerner-Smidt P, Scheutz F, Moller NE, On SL, Molbak K. Water-borne *Campylobacter jejuni* infection in a Danish town—a 6-week continuous source outbreak. Clin Microbiol Infect. 1998;4:648–56.
53. Godoy P, Artigues A, Nuin C, Aramburu J, Perez M, Dominguez A, et al. Outbreak of gastroenteritis caused by Campylobacter jejuni transmitted through drinking water. Med Clin (Barc). 2002;119:695–8.
54. Hanninen ML, Haajanen H, Pummi T, Wermundsen K, Katila ML, Sarkkinen H, et al. Detection and typing of *Campylobacter jejuni* and *Campylobacter coli* and analysis of indicator organisms in three waterborne outbreaks in Finland. Appl Environ Microbiol. 2003;69:1391–6.
55. Jones IG, Roworth M. An outbreak of *Escherichia coli* O157 and campylobacteriosis associated with contamination of a drinking water supply. Public Health. 1996;110:277–82.
56. Holme R. Drinking water contamination in Walkerton, Ontario: positive resolutions from a tragic event. Water Sci Technol. 2003;47:1–6.
57. Maurer AM, Sturchler D. A waterborne outbreak of small round structured virus, campylobacter and shigella co-infections in La Neuveville, Switzerland, 1998. Epidemiol Infect. 2000;125:325–32.
58. Melby K, Gondrosen B, Gregusson S, Ribe H, Dahl OP. Waterborne campylobacteriosis in northern Norway. Int J Food Microbiol. 1991;12:151–6.
59. Miettinen IT, Zacheus O, von Bonsdorff CH, Vartiainen T. Waterborne epidemics in Finland in 1998–1999. Water Sci Technol. 2001;43:67–71.
60. Sacks JJ, Lieb S, Baldy LM, Berta S, Patton CM, White MC, et al. Epidemic campylobacteriosis associated with a community water supply. Am J Public Health. 1986;76:424–8.
61. Duke LA, Breathnach AS, Jenkins DR, Harkis BA, Codd AW. A mixed outbreak of cryptosporidium and campylobacter infection associated with a private water supply. Epidemiol Infect. 1996;116:303–8.
62. Furtado C, Adak GK, Stuart JM, Wall PG, Evans HS, Casemore DP. Outbreaks of waterborne infectious intestinal disease in England and Wales, 1992–5. Epidemiol Infect. 1998;121:109–19.
63. Melby KK, Svendby JG, Eggebo T, Holmen LA, Andersen BM, Lind L, et al. Outbreak of *Campylobacter* infection in a subartic community. Eur J Clin Microbiol Infect Dis. 2000;19:542–4.
64. Rautelin H, Koota K, von Essen R, Jahkola M, Siitonen A, Kosunen TU. Waterborne *Campylobacter jejuni* epidemic in a Finnish hospital for rheumatic diseases. Scand J Infect Dis. 1990;22:321–6.
65. Stehr-Green JK, Nicholls C, McEwan S, Payne A, Mitchell P. Waterborne outbreak of *Campylobacter jejuni* in Christchurch: the importance of a combined epidemiologic and microbiologic investigation. N Z Med J. 1991;104:356–8.
66. Centers for Disease Control and Prevention. Outbreak of *Escherichia coli* O157:H7 and *Campylobacter* among attendees of the Washington County Fair—New York, 1999. MMWR Morb Mortal Wkly Rep. 1999;48:803–5.
67. Bopp DJ, Sauders BD, Waring AL, Ackelsberg J, Dumas N, Braun-Howland E, et al. Detection, isolation, and molecular subtyping of *Escherichia coli* O157:H7 and *Campylobacter jejuni* associated with a large waterborne outbreak. J Clin Microbiol. 2003;41:174–80.
68. Millson M, Bokhout M, Carlson J, Spielberg L, Aldis R, Borczyk A, et al. An outbreak of *Campylobacter jejuni* gastroenteritis linked to meltwater contamination of a municipal well. Can J Public Health. 1991;82:27–31.
69. Aho M, Kurki M, Rautelin H, Kosunen TU. Waterborne outbreak of *Campylobacter* enteritis after outdoors infantry drill in Utti, Finland. Epidemiol Infect. 1989;103:133–41.
70. Merritt A, Miles R, Bates J. An outbreak of *Campylobacter* enteritis on an island resort, north Queensland. Commun Dis Intell. 1999;23:215–9.
71. Said B, Wright F, Nichols GL, Reacher M, Rutter M. Outbreaks of infectious disease associated with private drinking water supplies in England and Wales 1970–2000. Epidemiol Infect. 2003;130:469–79.

72. Gillespie IA, O'Brien SJ, Frost JA, Adak GK, Horby P, Swan AV, et al. A case-case comparison of *Campylobacter coli* and *Campylobacter jejuni* infection: a tool for generating hypotheses. Emerg Infect Dis. 2002;8:937–42.
73. Moore J, Caldwell P, Millar B. Molecular detection of *Campylobacter* spp. in drinking, recreational and environmental water supplies. Int J Hyg Environ Health. 2001;204:185–9.
74. Moore JE, Caldwell PS, Millar BC, Murphy PG. Occurrence of *Campylobacter* spp. in water in Northern Ireland: implications for public health. Ulster Med J. 2001;70:102–7.
75. Savill MG, Hudson JA, Ball A, Klena JD, Scholes P, Whyte RJ, et al. Enumeration of *Campylobacter* in New Zealand recreational and drinking waters. J Appl Microbiol. 2001;91:38–46.
76. Hernandez J, Fayos A, Alonso JL, Owen RJ. Ribotypes and AP-PCR fingerprints of thermophilic campylobacters from marine recreational waters. J Appl Bacteriol. 1996;80:157–64.
77. Minaev VI, Cherkasskii BL, Volokhovich TT, Minaeva NZ, Pertin OS, Gorelov AV, et al. The leading pathways and factors in the transmission of the causative agents of campylobacteriosis under current conditions. Zh Mikrobiol Epidemiol Immunobiol. 1995;(2):39–42.
78. Goossens H, Giesendorf BA, Vandamme P, Vlaes L, Van den BC, Koeken A, et al. Investigation of an outbreak of *Campylobacter upsaliensis* in day care centers in Brussels: analysis of relationships among isolates by phenotypic and genotypic typing methods. J Infect Dis. 1995;172:1298–305.
79. Vandamme P, Pugina P, Benzi G, Van Etterijck R, Vlaes L, Kersters K, et al. Outbreak of recurrent abdominal cramps associated with *Arcobacter butzleri* in an Italian school. J Clin Microbiol. 1992;30:2335–7.
80. Morooka T, Umeda A, Fujita M, Matano H, Fujimoto S, Yukitake K, et al. Epidemiologic application of pulsed-field gel electrophoresis to an outbreak of *Campylobacter fetus* meningitis in a neonatal intensive care unit. Scand J Infect Dis. 1996;28:269–70.
81. Evans SJ. The seasonality of canine births and human campylobacteriosis: a hypothesis. Epidemiol Infect. 1993;110:267–72.
82. Ellis A, Irwin R, Hockin J, Borczyk A, Woodward D, Johnson W. Outbreak of *Campylobacter* infection among farm workers: an occupational hazard. Can Commun Dis Rep. 1995;21:153–6.
83. Schouls LM, Reulen S, Duim B, Wagenaar JA, Willems RJ, Dingle KE, et al. Comparative genotyping of *Campylobacter jejuni* by amplified fragment length polymorphism, multilocus sequence typing, and short repeat sequencing: strain diversity, host range, and recombination. J Clin Microbiol. 2003;41:15–26.
84. Nielsen EM, Engberg J, Fussing V, Petersen L, Brogren CH, On SL. Evaluation of phenotypic and genotypic methods for subtyping *Campylobacter jejuni* isolates from humans, poultry, and cattle. J Clin Microbiol. 2000;38:3800–10.
85. Savill M, Hudson A, Devane M, Garrett N, Gilpin B, Ball A. Elucidation of potential transmission routes of *Campylobacter* in New Zealand. Water Sci Technol. 2003;47:33–8.
86. Stanley K, Jones K. Cattle and sheep farms as reservoirs of *Campylobacter*. J Appl Microbiol. 2003;94(Suppl):S104–13.
87. Jones K, Howard S, Wallace JS. Intermittent shedding of thermophilic campylobacters by sheep at pasture. J Appl Microbiol. 1999;86:531–6.
88. Mann DR, Akinbami MA, Gould KG, Ansari AA. Seasonal variations in cytokine expression and cell-mediated immunity in male rhesus monkeys. Cell Immunol. 2000;200:105–15.
89. Stanley KN, Wallace JS, Currie JE, Diggle PJ, Jones K. Seasonal variation of thermophilic campylobacters in lambs at slaughter. J Appl Microbiol. 1998;84:1111–6.
90. Stanley KN, Wallace JS, Currie JE, Diggle PJ, Jones K. The seasonal variation of thermophilic campylobacters in beef cattle, dairy cattle and calves. J Appl Microbiol. 1998;85:472–80.
91. Willis WL, Murray C. *Campylobacter jejuni* seasonal recovery observations of retail market broilers. Poult Sci. 1997;76:314–7.
92. Srour SF, Rishpon S, Rubin L, Warman S. An outbreak of *Campylobacter jejuni* enteritis after farm visit in Haifa subdistrict. Harefuah. 2002;141:683–4.
93. Beecham HJ III, Lebron CI, Echeverria P. Short report: impact of traveler's diarrhea on United States troops deployed to Thailand. Am J Trop Med Hyg. 1997;57:699–701.
94. Mattila L, Siitonen A, Kyronseppa H, Simula I, Oksanen P, Stenvik M, et al. Seasonal variation in etiology of travelers' diarrhea. Finnish-Moroccan Study Group. J Infect Dis. 1992;165:385–8.
95. Pearson AD, Healing TD. The surveillance and control of campylobacter infection. Commun Dis Rep CDR Rev. 1992;2:R133–9.
96. Beecham HJ III, Lebron CI, Echeverria P. Short report: impact of traveler's diarrhea on United States troops deployed to Thailand. Am J Trop Med Hyg. 1997;57:699–701.
97. Haberberger RL, Jr., Mikhail IA, Burans JP, Hyams KC, Glenn JC, Diniega BM, et al. Travelers' diarrhea among United States military personnel during joint American-Egyptian armed forces exercises in Cairo, Egypt. Mil Med. 1991;156:27–30.
98. Black RE. Epidemiology of travelers' diarrhea and relative importance of various pathogens. Rev Infect Dis. 1990;12(Suppl 1):S73–9.
99. Black RE. Pathogens that cause travelers' diarrhea in Latin America and Africa. Rev Infect Dis. 1986;8(Suppl 2):S131–5.
100. Brasseur D, Casimir G, Goyens P. *Campylobacter jejuni* and infantile traveller's diarrhoea. Eur J Pediatr. 1986;144:517–8.
101. Echeverria P, Blacklow NR, Sanford LB, Cukor GG. Travelers' diarrhea among American Peace Corps volunteers in rural Thailand. J Infect Dis. 1981;143:767–71.
102. Kapperud G, Lassen J, Ostroff SM, Aasen S. Clinical features of sporadic *Campylobacter* infections in Norway. Scand J Infect Dis. 1992;24:741–9.
103. Pazzaglia G, Bourgeois AL, Araby I, Mikhail I, Podgore JK, Mourad A, et al. Campylobacter-associated diarrhoea in Egyptian infants: epidemiology and clinical manifestations of disease and high frequency of concomitant infections. J Diarrhoeal Dis Res. 1993;11:6–13.
104. Bichile LS, Saraswati K, Popat UR, Nanivadekar SA, Deodhar LP. Acute *Campylobacter jejuni* enteritis in 385 hospitalised patients. J Assoc Physicians India. 1992;40:164–6.
105. Louis V, Russek-Cohen E, Rubinstein MI, O'Brien SJ, Pearson AD, Colwell RR. Seasonality of *Campylobacter* cases in England and Wales (1990–1999) [abstract]. Presented at the 103rd General Meeting of the American Society for Microbiology; 2003 May 17–22; Washington, DC.
